# Targeted insertion and reporter transgene activity at a gene safe harbor of the human blood fluke, *Schistosoma mansoni*

**DOI:** 10.1016/j.crmeth.2023.100535

**Published:** 2023-07-24

**Authors:** Wannaporn Ittiprasert, Max F. Moescheid, Cristian Chaparro, Victoria H. Mann, Thomas Quack, Rutchanee Rodpai, André Miller, Prapakorn Wisitpongpun, Watunyoo Buakaew, Margaret Mentink-Kane, Sarah Schmid, Anastas Popratiloff, Christoph G. Grevelding, Christoph Grunau, Paul J. Brindley

**Affiliations:** 1 Department of Microbiology, Immunology & Tropical Medicine, School of Medicine & Health Sciences, George Washington University, Washington, DC 20037, USA; 2 Institute of Parasitology, Biomedical Research Center Seltersberg, Justus Liebig University Giessen, Giessen, Germany; 3 IHPE, University of Perpignan Via Domitia, CNRS, IFREMER, University Montpellier, Perpignan, France; 4 Department of Parasitology and Excellence in Medical Innovation, and Technology Research Group, Faculty of Medicine, Khon Kaen University, Khon Kaen 40002, Thailand; 5 Schistosomiasis Resource Center, Biomedical Research Institute, Rockville, MD 20850, USA; 6 Faculty of Medical Technology, Rangsit University, Pathum Thani 12000, Thailand; 7 Department of Microbiology, Faculty of Medicine, Srinakharinwirot University, Bangkok 10110, Thailand; 8 Nanofabrication and Imaging Center, Science & Engineering Hall, George Washington University, Washington, DC 20052, USA

**Keywords:** overlapping CRISPR target, reporter transgene, gene safe harbor, human blood fluke, *Schistosoma mansoni*

## Abstract

The identification and characterization of genomic safe harbor sites (GSHs) can facilitate consistent transgene activity with minimal disruption to the host cell genome. We combined computational genome annotation and chromatin structure analysis to predict the location of four GSHs in the human blood fluke, *Schistosoma mansoni*, a major infectious pathogen of the tropics. A transgene was introduced via CRISPR-Cas-assisted homology-directed repair into one of the GSHs in the egg of the parasite. Gene editing efficiencies of 24% and transgene-encoded fluorescence of 75% of gene-edited schistosome eggs were observed. The approach advances functional genomics for schistosomes by providing a tractable path for generating transgenics using homology-directed, repair-catalyzed transgene insertion. We also suggest that this work will serve as a roadmap for the development of similar approaches in helminths more broadly.

## Highlights

• Genome safe harbor sites predicted using genome annotation and chromatin structure
• Homology-directed insertion of transgene into schistosome genomic safe harbor
• Gene editing at schistosome genome safe harbor enhanced by multiple overlapping gRNAs
• Potential to advance functional genomic studies in schistosomes

## Motivation

Functional genomics methods are needed to advance the study of helminth parasites, which cause neglected tropical diseases of significant global health burden. The motivation for this work was to develop a tractable method for transgenic studies of the human blood fluke, *Schistosoma mansoni*, by identifying genome safe harbor sites and demonstrating successful homology-directed transgene insertion.

## Graphical abstract

**Figure:**
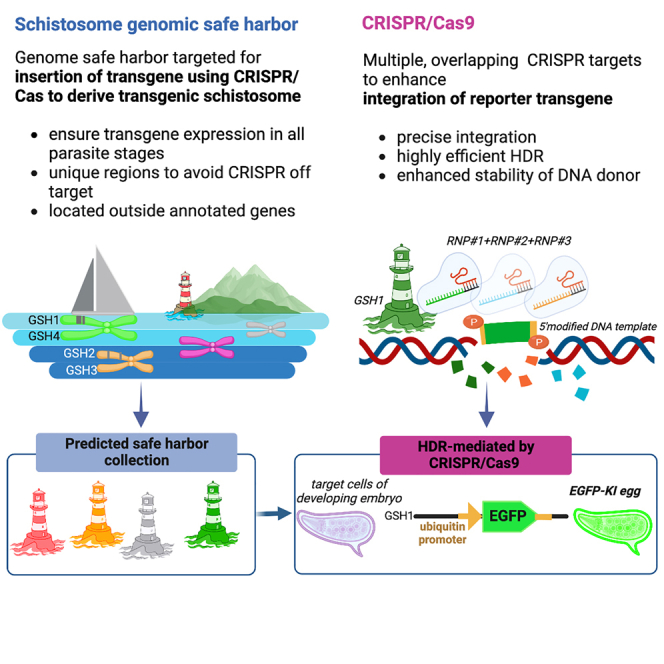

## Teaser

Approaches to generate tractable transgenics are needed for helminths responsible for major neglected tropical diseases. Ittiprasert et al. computationally identify genomic safe harbor sites and successfully insert a reporter transgene in one of them, demonstrating tractable transgenic studies in the human blood fluke, *Schistosoma mansoni.*

## Introduction

Clustered regularly interspaced short palindromic repeats (CRISPR) technology has revolutionized functional genomics.^1^^,^^2^^,^^3^ Transgenesis approaches are integral in diverse applications including therapeutics and deciphering host-pathogen interactions. With progress emanating from model species and cell lines, tools can frequently be adapted and transferred to non-model species. Among these are the helminths responsible for major neglected tropical diseases, which cause substantial morbidity and mortality.^4^ Infections with helminths also are responsible for substantial economic and disease burdens in agriculture and animal health.^5^ These public health and economic imperatives motivated international collaboration for parasite *omics* research that has resulted in outsized databases of genomes and proteomes and gene, transcript, and protein annotations.^6^^,^^7^^,^^8^^,^^9^ In the current post-genomics era, and despite the availability of these omics data, tools for functional genomics in parasitic helminths have been limited to RNAi, which performs with variable efficacy.^10^^,^^11^ Therefore, CRISPR-based transgenesis protocols for functionally characterizing genes of interest such as those coding for putative drug and/or vaccine targets are a prominent research priority. Moreover, progress with gene editing in schistosomes will facilitate its use in other major invertebrate clades of the Protostomia, including the planarians, for which CRISPR-based genetics have yet to be reported.

CRISPR enables targeted site-specific mutation(s), obviating an impediment of earlier transgenesis approaches that relied on vector-based particle bombardment,^12^ lentiviruses,^13^ and transposons such as *piggyBac*.^14^ These latter approaches could lead to genetic instability, multi-copy insertion, or inactivation of the transgene and interference with the endogenous gene under investigation. These issues can be overcome in the process of genome editing, where double-stranded breaks (DSBs) are resolved by several discrete repair mechanisms, particularly the predominant error-prone, non-homology end joining (NHEJ) pathway and by homology-directed repair (HDR). HDR efficiency can be improved when supplied with double-strand (ds) DNA donor with modifications as the repair template.^15^ CRISPR-Cas-assisted HDR has been applied in *Schistosoma mansoni*^16^^,^^17^ with a promoter-free, single-strand deoxynucleotide. Overlapping CRISPR target sites improve precise HDR insertion in embryonic stem cells,^18^^,^^19^ with modification of 5′ termini of long dsDNA donors, bolstering efficient, single-copy integration through the retention of a monomeric donor confirmation and thereby enabling gene replacement and tagging.^20^

One of the caveats of transgene integration is that transgene insertion into an arbitrarily chosen position in the genome may lead to loss of expression due to disruption of cell function or repressive chromatin structure in the target region. This had been identified as a major drawback, initially in gene therapy approaches, and it has led to the concept of genome safe harbors (GSHs).^21^^,^^22^^,^^23^^,^^24^ An ideal GSH has been defined as a region (1) that does not overlap (predicted) functional DNA elements and (2) that lacks heterochromatic marks that could impede transcription.^25^ This approach was successfully used in *Caenorhabditis elegans* based on annotations from the ENCODE and modENCODE consortia.^26^ For non-model organisms, chromatin structure annotations are often unavailable, and experiments resort to criterion (1). For instance, transgene insertion into a GSH of the human filarial parasite *Brugia malayi* has been reported, but in that case, GSHs were predicted based on four sequence annotation features alone: to be located in intergenic regions, to be unique in the genome, to contain a terminal protospacer adjacent motif (PAM) necessary for targeting by the sgRNA-Cas9 ribonucleoprotein complex (RNP), and fourth, the putative PAM is admissible only if situated >2 kb from the nearest predicted coding region.^27^

In this study, we profited from the availability of chromatin data and chromatin accessibility data and combined them in a computational investigation with genome sequence information to identify potential GSH sites in *S. mansoni*. Furthermore, we adapted CRISPR-Cas9-based approaches to insert a reporter transgene into the most qualified out of four predicted candidate GSHs. The donor transgene encoded EGFP under the control of the schistosome ubiquitin gene promoter and terminator. The targeted region was free of repetitive sequences and neighboring long non-coding regions, a situation likely to minimize off-target CRISPR-Cas activity. Multiple sites within this region were targeted with overlapping guide RNAs, deployed in unison to enhance editing efficiency and HDR in the presence of the phosphorothioate-modified DNA donor. A knockin (KI) efficiency of 75% was observed for expression of EGFP in miracidia developing within the schistosome eggshell.

## Results

### GSHs predicted in the schistosome genome

**Figure 1:**
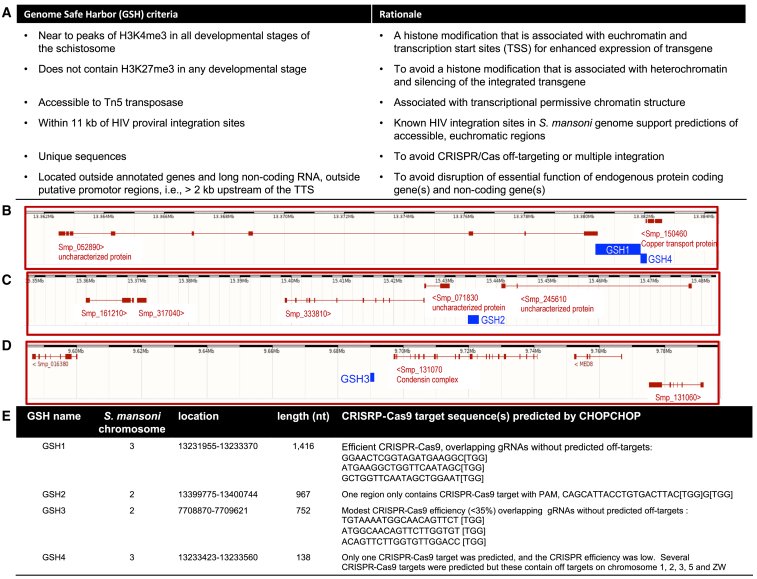
Identification of GSHs and their CRISPR targets (A) Criteria and rationale used to computationally predict GSHs. (B–D) Chromosomal locations and lengths of four candidate GSH sites (blue boxes), located on chromosome 2 (GSH2, GSH3) and chromosome 3 (GSH1, GSH4) of *S. mansoni* genome v9. All criteria were equally weighted. In addition, coding sequences of genes identified in the vicinity of the four (gene-free) GSHs are shown, which were mostly uncharacterized proteins (red boxes with connected red line). Black and white scale bars at the top of each panel represent 1 Mb in length. (E) Features of the GSHs including chromosomal location, length, CRISPR-Cas9 targets, gRNAs, and their predicted on- and off-target specificities by CHOPCHOP tool.

To identify potential GSH sites, we performed *in silico* analyses based on accepted criteria, introduced principles,^28^ and genome resources for *S. mansoni*, which could satisfy benign and stable gene expression. Notably, we sought to identify intergenic GSH, rather than intragenic GSH.^28^ Four regions satisfied our criteria (Figure 1) and were termed GSH1 (1,416 bp; location, chromosome 3:13380432–13381848), GSH2 (970 bp; chromosome 2:15434976–15435954), GSH3 (752 bp; chromosome 2:9689988–9690739), and GSH4 (138 bp; chromosome 3:13381901–13382038), respectively. We note that several protein-coding loci were situated in the vicinity of these gene-free GSHs, although these genes were >2 kb distant from any GSH: Smp_052890, uncharacterized protein; Smp_150460, copper transport protein; Smp_071830, uncharacterized protein; Smp_245610, uncharacterized protein; and Smp_131070, condensing complex (Figures 1B–1D). Most of these genes are as yet uncharacterized proteins and may be non-essential genes based on orthology to essential genes known from eukaryotes.^29^ For CRISPR-specific considerations for the programmed transgene insertion, particularly the presence of multiple PAMs, GSH1 qualified as the most useful of the four GSHs for the present investigation, and hence programmed gene editing at GSH1 is the focus of the findings detailed below.

### Efficiency of programmed mutation at GSH1 enhanced by multiple gRNAs

**Figure 2:**
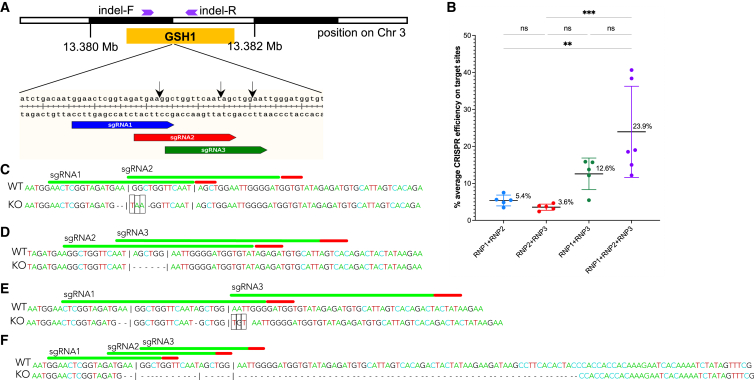
Efficiency of NHEJ enhanced using overlapping gRNAs (A) Schematic map of sites of the overlapping gRNAs (blue, red, and green arrows for target 1, 2, and 3, respectively) within GSH1 (yellow box), along with primer locations for indel analysis (purple arrows). The black arrows indicate the DSB programmed by sgRNA1, 2, and 3. (B) Efficiency of CRISPR at GSH1 in eggs of *S. manson*i, as assessed with the DECODR algorithm using distance, following transfection with overlapping gRNPs: RNP1+RNP2 (blue dots), RNP2+RNP3 (red), RNP1+RNP3 (green), and RNP1+RNP2+RNP3 (purple). Significantly higher CRISPR efficiency was obtained with the three overlapping gRNPs, mean = 23.6%, than the other groups (p ≤ 0.001). Among the groups transfected with dual RNPs, efficiency obtained with the RNP1+RNP3 treatment group, mean = 12.6%, was significantly higher than either of the other groups, RNP1+RNP2 at 5.4% and RNP2+RNP3 at 3.6% (p ≤ 0.01; one-way ANOVA with 95% confidence intervals, six biological replicates; GraphPad Prism). (C–E) Representative alleles, in the schistosome genome, bearing indels at the target site in GSH1 following transfection with dual RNPs, as a gauge of efficiency in CRISPR-catalyzed gene editing. The reference WT allele is shown above the KO allele. KOs were identified for small deletions, 1–6 nt in length (dash) or insertion/substitution (black boxes). The vertical black line boxes show PAM sites. (F) A representative example of a KO allele bearing a large-sized deletion resulting from transfection with the triple gRNPs.

We proceeded to investigate the efficiency of programmed mutation and reporter transgene activity at GSH1. Overlapping gRNAs were employed, an approach that enhanced KI efficiency in mammalian cell lines and embryos.^18^^,^^19^ Among the gRNAs exhibiting on-target specificity for GSH1, three overlapping gRNAs (sgRNA1, sgRNA2, and sgRNA3), which lacked self-complementarity and off-target matches to *S. mansoni* genome (Figures 1B, 1E, and 2A), were selected from among CRISPR-Cas9 target sites.^30^^,^^31^ The RNPs of Cas9 nuclease and sgRNA were assembled, after which four discrete mixtures of RNPs were used. Three of the mixtures included dual RNPs (RNP1+RNP2, RNP2+RNP3, and RNP1+RNP3), and the fourth included the triple RNPs (RNP1+RNP2+RNP3).

The mixtures of multiple RNPs, along with the DNA donor encoding EGFP, were co-electroporated into schistosome eggs. The transfected eggs were cultured for 15 days, after which EGFP expression was quantified. Efficiency of genome editing, both in controls and experimental groups, was assessed using DECODR^32^ analysis of Sanger sequence chromatograms of amplicons that spanned the DSBs. Analysis of PCR products from DNA using indel primers flanking the DSBs (Figure 2A) revealed knockout (KO) efficiency, as assessed by indel (insertions/deletions)-bearing alleles resulting from the dual gRNAs, as follows: KO frequencies at GSH1 of 5.4% (0.8%–10.4%), 3.6% (1.2%–19.3%), and 12.6% (4.9%–19.3%) for RNP1+RNP2, RNP2+RNP3, and RNP1+RNP3, respectively (Figure 2B). The dual RNPs induced short deletions of one to several nucleotides at the predicted DSB for sgRNA1, 2, and/or 3 (Figures 2C and 2E). Mutations were not evident in amplicons from the control groups. The triple RNPs resulted in 23.9% KO (2.4%–71.9%), higher than achieved with any mixture of the dual RNPs (Figure 2B).

The CRISPR efficiencies at each target site varied among the RNP mixtures (Figure S1A). KO efficiency at each target resulting from dual RNPs was generally lower than from triple RNPs. At target 1, KO rates were 6.9% (4.1%–10.4%), 13.8% (6%–19.3%), and 26.5% (14%–33.2%) using RNP1+RNP2, RNP1+RNP3, and RNP1+RNP2+RNP3, respectively. There were 3.9% (0.8%–5.8%), 1.8% (1.2%–2.4%), and 14.2% (2.4%–16.8%) mutations on target 2 using RNP1+RNP2, RNP2+RNP3, and RNAP1+RNP2+RNP3, respectively. Mutation efficiencies at target 3 using RNP2+RNP3 and RNP1+RNP3 were 13.9% (6%–19.3%) and 11.3% (6%–19.3%). The mutational profiles of the indels were mostly deletions rather than insertions (Figures S1B–S1E). Conspicuously, deletions up to 115 nt were identified with the triple RNPs (Figures 2E and S1E). KO efficiency was assessed using at least five biological replicates. Combining three overlapping gRNAs induced an aggregate mutation efficiency (23.9%) higher than that obtained with any of the dual RNPs: 5.4%, 3.6%, 12.6% (p ≤ 0.001, one-way ANOVA). The KO efficiency of the RNP1+RNP3 group was higher than that of either of the other dual RNPs (p ≤ 0.01) (Figure 2B).

### Overlapping gRNAs enhanced efficiency of CRISPR knockin

**Figure 3:**
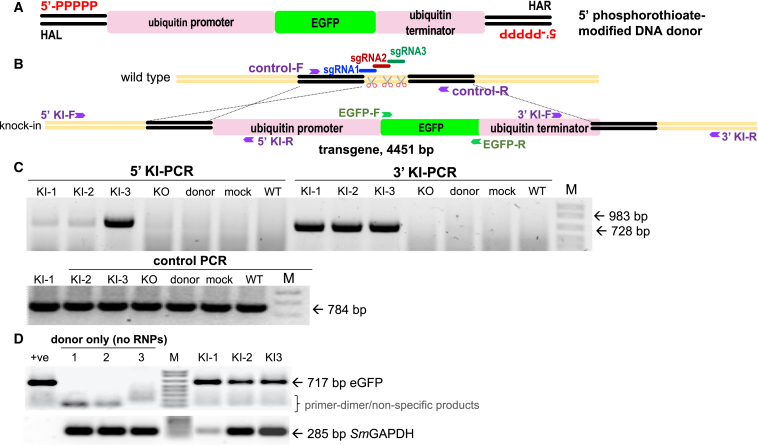
Targeted transgene insertion and expression in the schistosome egg Programmed CRISPR-Cas9 KI of a donor template of 4.451 kb in length, encoding EGFP driven by ubiquitin promoter and terminator. (A) Topology of lsDNA donor prepared from a primer pair with 5′-5×-phosphorothioate modification. The donor encoded the ubiquitin promoter (pink bar) driving EGFP (green) and ubiquitin terminator (pink) and was flanked at its termini with ∼600-bp HAs (black bars). The HAL was situated at the position of sgRNA1, and the HAR was downstream of the prototypic adjacent motif of sgRNA 3. (B) Illustration of the WT and KI alleles after DSBs programmed by the overlapping sgRNAs 1, 2, and 3. PCR primers are indicated by the purple arrows. (C) Targeted KI of the transgene detected by PCR using 5′ KI (983 bp) or 3′ KI (728 bp) primers. Negative controls for KI included WT, mock, donor-only treatment, or KO groups. Lanes KI-1, KI-2, and KI-3 show amplicons from three biological replicates of KI of the transgene. Other lanes show the outcome of RT-PCRs with donor electroporation (no CRISPR materials). The integrity of the DNAs was confirmed by the presence of the amplicon of 784 bp in all lanes of the gel labeled “control PCR.” Primer-dimer and/or non-specific PCR band(s) from donor transfected eggs were seen ≤100 bp in size. (D) Expression of EGFP transcript; 717 bp as assessed by RT-PCR following programmed KI of the transgene into GSH1. The integrity of the RNAs was assessed by analysis of transcripts of the reference gene, *Sm*GAPDH, with an expected amplicon of 285 bp. DNA donor was used as the positive PCR template. Transcription of *Sm*GAPDH was seen in all treatment and control groups (bottom panel) but not in the donor-only group.

As multiple gRNAs with overlapping sequences can enhance CRISPR-Cas9-mediated HDR efficiency^18^ and given that triple overlapping gRNAs performed better than dual gRNAs in initiating programmed mutation at GSH1 in eggs (Figure 2B), we investigated KI of a reporter transgene at GSH1 with the triple overlapping sgRNA/RNPs (Figures 3A and 3B). We employed the gene encoding EGFP driven by the promoter of the endogenous *S. mansoni* ubiquitin gene (Smp_335990) and its cognate terminator region as the repair template for programmed HDR (Figure 3A). The donor template included homology arms (HAs) specific for GSH1, located on the 5′-flanking region of target 1 and the 3′-flanking region of target 3 (Figure 3B). The donor template was delivered as linearized, long, double-stranded DNA (lsDNA) of 4,451 bp in length. Aiming for precise and efficient single-copy integration of the donor transgene into GSH1 by HDR, the 5′ termini of the DNA donor amplicons were chemically modified^20^ to shield the donor template from multimerization and from integration at the DSB via the NHEJ repair pathway (Figure 3A).

At the outset, we investigated the impact of length of the HA by comparing the performance of the donor template bearing HAs of increasing length of 200, 400, and 600 bp. Dual (RNP1+RNP3) and triple (RNP1+RNP2+RNP3) RNP mixtures were used in this investigation. EGFP expression was not evident in eggs electroporated with lsDNA donors with 200- and 400-bp HAs at 5 days after transfection (not shown). By contrast, we observed a few EGFP-positive eggs (∼2%–3% with at least a small number of EGFP-expressing cells; data from four biological replicates) with the lsDNA donor with 600-bp HA (not shown). Subsequently, we focused the investigation for EGFP expression on transfection with the donor transgene flanked by 600-bp HA using the triple RNPs and monitored EGFP expression for up to 15 days. Thereafter, on examination using spectrally resolved, confocal laser scanning microscopy (CLSM), the EGFP signals were detected in the eggs of the experimental group, which received the CRISPR materials including the lsDNA donor with 600-bp HA. EGFP signals remained until 15 days, when the experiment ended. EGFP signals were not observed in the negative control groups, although the autofluorescence characteristic of schistosome eggs was apparent.^33^ EGFP signals were also detected in the lsDNA donor control (without RNPs) for several days, indicating that extrachromosomal lsDNA expressed EGFP transiently after the transfection.

Next, we investigated the programmed KI by PCR-based analysis for the presence of the expected amplicons spanning the 5′ and 3′ flanks of the donor transgene, i.e.*,* flanking the ubiquitin promoter, EGFP, and the ubiquitin terminator sequences. At the 5′-flanking region, we used a forward primer specific for the genome upstream of the 5′ HA paired with a reverse primer specific for the ubiquitin promoter (Figure 3B). For the 3′ integration junction, a reverse primer specific for a site downstream of the 3′ terminus of the HA paired with a forward primer specific for the ubiquitin terminator was used. Fragments representing the 3′ KI and 5′ KI integration regions of 983 bp and 728 bp, respectively, were observed in the treatment groups but not in the control groups (Figure 3C). EGFP transcripts were observed in the KI experimental group, although some variability in transcript abundance among the biological replicates was seen based on the signals obtained for *Sm*GAPDH, which served as the reference gene (Figure 3D).

### Reporter transgene expression in edited eggs

**Figure 4:**
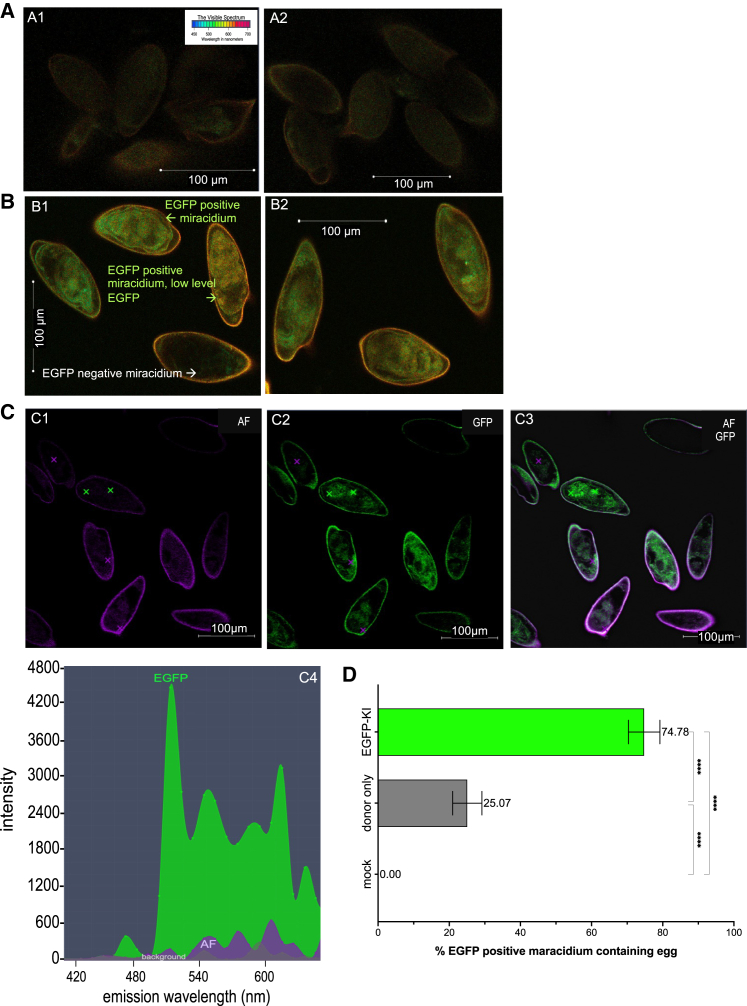
More EGFP-positive eggs following programmed KI at GSH1 (A) Confocal laser scanning micrographs: eggs exhibiting autofluorescence background (AF) from the control group, i.e.*,* eggs transfected only with the DNA; A1 and A2 are representative images from biological replicates. (B) Eggs expressing EGFP in the KI group transfected with triple RNPs and DNA donor; B1 and B2 are representative images from two biological replicates. Eggs expressed EGFP with a broad range in intensity of EGFP ranging from higher (green arrow) and lower levels (yellow arrow) following programmed KI. Eggs expressed EGFP, as observed from day 5 until day 15 when the experiment was terminated. (C) Linear unmixing analysis revealed EGFP in miracidia, as shown in these representative micrographs (C1, C2 and C3, C4), and plot showing the emission spectrum of EGFP (green curve) with a peak signal at 509 nm (× in bright green indicates the EGFP signal, while the × in purple color indicates the AF inside the egg [purple curve]). (D) To assess expression of EGFP, eggs were scored as positive when >20% of the miracidium surface area was EGFP positive; representative EGFP-positive eggs are shown in panels C2 and C3. On day 15 following transfection, eggs were scored and counted for EGFP positivity in each of four biological replicates (∼100 eggs/replicate) of KI and lsDNA-only eggs. About 25% of the eggs in the lsDNA-only group were scored as EGFP positive (19%–32%), whereas 75% of eggs in the KI group expressed EGFP (68%–79%); p < 0.001; two-tailed t = 69.87, df = 142; difference between means (EGFP-KI-only donor) ± SEM, 49.7 ± 0.7, 95% CI, 48.3–51.1.

EGFP positivity and intensity were quantified using spectral laser scanning CLSM.^33^ Active transgene expression was confirmed within miracidia developing inside transfected eggs (Figures 4A and 4B). EGFP appeared to be expressed by numerous diverse cells throughout the developing larvae, whereas morphological malformation was not observed in transgenic eggs and their enclosed larvae. More intense EGFP fluorescence was consistently recorded and quantified at 509 nm in eggs from the experimental treatment group (Figures 4B1 and 4B2) than the mock eggs and in eggs transfected solely with donor template (Figures 4A1 and 4A2). Subsequently, on day 15 following transfection with the repair template in the presence or absence of the RNPs mixture, we quantified EGFP intensity in eggs that contained a miracidium by normalization with the EGFP signal from lsDNA-only transfected eggs. Fluorescence intensity differed markedly between these two groups: by 15 days after transfection, 75% of miracidia in eggs from the KI group emitted EGFP fluorescence, whereas 25% of eggs containing a miracidium transfected with the lsDNA donor only emitted EGFP (Figures 4C and 4D) (p ≤ 0.001; n = 402 eggs in the experimental group, n = 397 eggs in the lsDNA-only group; Figure 4E; collected from four independent biological replicates).

In addition, we scored the intensity of fluorescence at 509 nm,^33^ the emission wavelength for EGFP, as shown in Figures 4C2, 4C3, and 4D (green curve). To this end, we subtracted the signal at 509 nm from the autofluorescence spectrum, which originated from the eggshell. The EGFP-specific signal in the control lsDNA donor repair template treatment group, mean = 1,290 arbitrary units (AU)^34^ (range, 856–11,713), was significantly lower than the experimental group transfected with the triple, overlapping guide RNPs mixture in the presence of the donor repair template, 6,905 AU (range, 4,973–8,963) (p ≤ 0.001) (Figure S2). Moreover, emission of EGFP was not detected in the control groups, i.e.*,* mock-transfected and WT eggs (not shown). Diverse cells and tissues of the developing miracidium expressed the fluorescence reporter gene, so EGFP expression appeared not be restricted to specific cells (Figures 4B and 4C).

### Impact on egg viability by electroporation

During the investigation, we also examined delivery of CRISPR materials using electroporation of the schistosome egg, an approach originally described for transfection of the schistosomulum stage of *S. mansoni*.^35^ At the outset, electroporation voltage was investigated, using a single pulse of 20 ms duration of 125, 150, 200 and 250 V to deliver the RNPs and donor template into the eggs. Thereafter, the eggs were cultured, and miracidial hatching was assessed 7 days later. Survival and/or larval growth inside the egg in the 125-V treatment group was not significantly affected; the rates of miracidial hatching were 26.6% ± 3.2% and 31.9% ± 2.6% from the non-electroporated group (Figure S3A). By contrast, increasing the voltage negatively impacted hatching of miracidia from the eggs: 150 V, 22.8% ± 2.2%; 200 V, 11.4% ± 1.2%; 250 V, 3.9% ± 2.0%, respectively (p < 0.001, two-way ANOVA) (Figure S3A). Last, we investigated the impact of the CRISPR materials in addition to voltage. Using electroporation at 125 V, we monitored hatching in two biological replicates. In the first, 41.2% ± 2.1%, 39.5% ± 1.5%, and 40.9% ± 1.9% (mean ± SE) of miracidia hatched from the wild-type (WT), donor-transfection-only, and experimental (EGFP KI) groups, and in the second were 59.1% ± 2.4%, 60.5% ± 0.6%, and 60.1% ± 1% from WT, donor-transfection-only, and EGFP KI groups, respectively (Figures S3B and S3C).

## Discussion

To advance functional genomics for helminths, we identified four potential GSH sites in *S. mansoni*, optimized conditions for delivery and structure of transgene cargo, and inserted the reporter transgene into the most qualified intergenic GSH1 by programmed CRISPR-Cas9 HDR repair. We confirmed integration of the transgene by amplicon sequencing as well as EGFP reporter activity using RT-PCR and CLSM analyses. Our approach for programmed editing in this helminth involved electroporation-based delivery to the schistosome egg of RNPs with overlapping gRNAs in the presence of phosphorothioate-modified, double-stranded donor targeting at GSH1. The procedure yielded 24% editing efficiency that was accompanied by transgene activity in 75% of miracidia in the genome-edited schistosome eggs. The donor dsDNA encoded the EGFP gene driven by the schistosome ubiquitin promoter and terminator. Furthermore, clear EGFP signals indicated the suitability of the regulatory elements of the ubiquitin gene to induce transgene expression.

This methodical approach provides a tractable path toward transgenic helminths using HDR-catalyzed transgene insertion. Our criteria to predict GSH included location in euchromatin to avoid silencing of the transgene, a unique genome-target sequence to minimize off-target events, avoidance of lncRNA-encoding genes, presence of epigenetic marks for open chromatin structure, and the absence of epigenetic marks indicating heterochromatin. We termed the intergenic sites GSH1, -2, -3, and -4, which were located on chromosomes 2 and 3. *S. mansoni* has seven pairs of autosomes and one pair of sex chromosomes, Z and W, with the female schistosome being the heterogametic sex.^36^ In addition, we assessed the GSH1 locus for CRISPR-Cas9 integration, gene editing, and overexpression of EGFP. We edited GSH1 using RNP of Cas9 endonuclease with multiple overlapping gRNAs. Triple RNPs delivered significantly higher CRISPR-Cas9 efficiency than dual RNPs and longer length deletion mutations. In addition, efficient HDR was obtained using a combination of multiple and overlapping RNPs programmed to cleave GSH1 in the presence of a repair template protected by chemical modifications. Our approach successfully inserted an lsDNA (4,551 bp) at GSH1. This outcome aligns with reports in cell lines and rodents involving overlapping gRNAs, where deletions close to the targeted mutation enhanced the efficiency of HDR.^18^^,^^37^ Overlapping gRNAs rather than simply multiple gRNA may be more efficient for gene KO in *S. mansoni* given recent findings involving CRISPR interference that compared both single and multiple gRNAs.^18^^,^^19^^,^^38^

GSH1 represents a promising CRISPR target for *S. mansoni*. Notably, 75% of eggs exhibited EGFP in the miracidium developing within the eggshell and significantly more fluorescence than seen in the control eggs transfected only with donor template. EGFP signals were not present in the control, untreated WT eggs, which by ∼10 days following transfection exhibited minimal background fluorescence. Our approach to evade the autofluorescence emitted by eggs, which can confound detection of EGFP, used spectral imaging and linear unmixing,^39^^,^^40^ an approach to facilitate quantification of EGFP-specific emission to resolve overlap between the EGFP and endogenous fluorophores in schistosomes. Eggs isolated from livers of *S. mansoni-*infected mice were co-electroporated with two or three RNPs and the donor transgene. Such a preparation of schistosome eggs includes eggs displaying a spectrum of development—from newly laid eggs containing the zygote, developing embryos, eggs containing the fully developed miracidium (with miracidial movement evident), and some dead eggs.^41^ Notably, however, the entry of active CRISPR materials and donor transgene into cells of each egg and each developmental stage cannot be predicted. The suitability of this approach for transfection of the LE population with RNPs has been demonstrated by RNP tracking analysis.^42^ Indeed, the outcome would be stochastic: not every egg would be expected to receive the full complement of RNPs and donor. Accordingly, eggs exhibiting minimal EGFP may not have been transfected as efficiently as eggs with stronger EGFP fluorescence.

Fluorescence throughout miracidial tissues was achieved using EGFP driven by the schistosomal ubiquitin promoter and terminator, emphasizing the ubiquitous activity of this gene as predicted by transcriptome analyses.^43^^,^^44^^,^^45^ This outcome confirmed reporter gene activity under the control of these ubiquitin elements and demonstrated the accessibility of GSH1 for the transcriptional machinery after programmed KI. The findings also revealed the feasibility of selection at the microscopic level, which would enable hand-picking of reporter-positive miracidia for snail infection to complete the life cycle. Following snail infection, fluorescing cercariae, or in the case of mono-miracidial infections reporter gene PCR-positive cercariae, could be selected for infection of laboratory rodents to propagate heritably transgenic worms. In future approaches, GSH1 may alternatively be used as a locus to integrate other transgenes, e.g., antibiotic resistance gene(s), to enable drug selection at the stage of embryogenesis or during the intermediate host stage in the snail. Here, oxamniquine is a suitable candidate drug.^42^ Where mono-miracidial infections with reporter gene-positive miracidia are performed, additional selection manipulations (microscopic or PCR based) could be undertaken on the clonal cercariae derived by this approach. Thereafter, reporter gene-positive female and male cercariae, in which gender can be confirmed by PCR,^46^ would enable genetic crosses in the mammalian host.^47^

These findings are consequential in that they advance functional genomics for a hitherto unmet challenge to manipulate a pathogen of global public health significance. They confirm that transgenes can be inserted into a predicted GSH to endow individual stages or populations of these pathogens with functions, with broad potential for basic and translational studies.^48^^,^^49^^,^^50^ Whereas this report deals with somatic transgenesis of the schistosome larva, the same approach is used for transfection of the newly laid egg (NLE) of *S. mansoni*, a stage that at its origin includes a single zygote (surrounded by vitelline yolk cells). The NLE represents a window to the germline, and the hypothesized accessibility of its zygote may facilitate complete transformation to derive lines of transgenic parasites carrying gain- or loss-of-function mutations. In addition, the gene editing methods developed here can be adapted for KO approaches of other genes of interest, in schistosomes, and likely other platyhelminths, for which genome sequences are available to be analyzed for GSHs. The information presented provided insights into efficient transgenesis and forward genetics for *S. mansoni* for other parasitic (and free-living) helminths.

### Limitations of the study

We focused on GSH1 because there were limitations to progress with the other prospective GSHs 2–4. The CHOPCHOP software predicted only a single CRISPR-Cas9 target in these three GSHs, and moreover, the site predicted in GSH4 was not specific and showed potential off-target hits elsewhere in the genome. For GSH3, CHOPCHOP predicted only low, <35%, editing efficiency. Overall, locating a Cas9 PAM^51^ is constrained by the AT-rich nature of the schistosome genome.^52^ Since this investigation deployed multiple overlapping gRNAs^18^ to facilitate homology-directed insertion of transgene, we ranked GSH1 as the most qualified for our purposes because multiple PAMs were present, along with the absence of off-target activity. Although a distance >2 kb from known genes was one of our criteria for GSHs in *S. mansoni*, intragenic sites rather than intergenic GSHs nonetheless may have expedient attributes for functional genomics where partial loss of fitness may be less consequential. Yet, intergenic sites are inherently safer given coding regions or other elements are not disrupted.

## STAR★Methods

### Key resources table

**Table:** 

REAGENT or RESOURCE	SOURCE	IDENTIFIER
**Chemicals, peptides, and recombinant proteins**		

*Streptococcus pyogenes* Alt-R HiFi Cas9 nuclease	IDT, Coralville, IA	Cat. No.#-1081058
DNA/RNA shield solution	Zymo Research, CA	Cat no. R1100
Percoll	MilliporeSigma	Cat no. P1644
Opti-MEM	ThermoFisher Scientific	Cat no. 31985062
DMEM	ThermoFisher Scientific	Cat no. 11320033
Fetal bovine serum	Gibco	Cat no. A5256801
Antibiotic antimycotic	Gibco	Cat no. 15240062
DMSO	ThermoFisher Scientific	Cas 67-68-5
Phusion® DNA polymerase	New England Biolabs, Ipswich, MA	Cat no. M0530
NucleoSpin Gel and PCR Cleanup and gel extraction kit	Takara, San Jose, CA	Cat no. 740609
RNAzol® RT	Molecular Research Center, Inc., Ciccinnati, OH	RN190
DNAzol® ES	Molecular Research Center, Inc., Ciccinnati, OH	DN 128
GoTaq® G2 DNA polymerase	Promega, Madison, WI	Cat no. M7841

**Deposited data**		

Gene Safe Harbor data	This paper	Zenodo Database: https://zenodo.org/record/7602535#.ZAIgqBPMLIM

**Experimental models: Organisms/strains**		

*S. mansoni* infected mice	Schistosomiasis Resource Center, Biomedical Reseach Institute, Rockville, MD	https://www.afbr-bri.org/schistosomiasis/

**Oligonucleotides**		

Custom guide RNA, see Figure 2A	IDT, Coralville, IA, https://www.idtdna.com/pages/products/crispr-genome-editing/alt-r-crispr-cas9-system	N/A
Primers for PCR	This paper	Table S1

**Recombinant DNA**		

pUC-Ubi-EGFP-ubi	This paper	N/A

**Software and algorithms**		

CHOPCHOP	http://chopchop.cbu.uib.no/	N/A
DECODR v3.0	https://decodr.org/	N/A
TIDE	https://tide.nki.nl/	N/A
RepeatModeler2 V2.0.1	http://www.repeatmasker.org/RepeatModeler/	N/A

**Other**		

NanoDrop One	ThermoFisher Scientific	Catalog number: ND-ONE-W
Electroporation cuvette	Cole-Parmer, BTX, Holliston, MA	Mfr # 45-0126
Electro SquarePorator	Cole-Parmer, BTX, Holliston, MA	ECM830
Disposable pestle and collection tube; BioMasher II (EOG-sterilized)	BioMasher II, Funakoshi, Diagnocine LLC, NJ	Code 320103
Power Masher II	BioMasher II, Funakoshi, Diagnocine LLC, NJ	Code 891300
Zeiss LSM710 Meta detector fitted Axiovert 200	Carl Zeiss, Jena, Germany	Model LSM710

### Resource availability

#### Lead contact

Further information and requests for resources and reagents should be directed to and will be fulfilled by the Lead Contact, Paul J Brindley: pbrindley@gwu.edu.

#### Materials availability

This study did not generate new unique reagents.

### Experimental model and study participant details

#### Mice

Mice (female, Swiss Webster) infected with *S. mansoni* were obtained from the Schistosomiasis Resource Center (Biomedical Research Institute, Rockville, MD) within seven days of infection by cercariae (180 cercariae/mouse/percutaneous infection). The mice were housed at the Animal Research Facility of George Washington University, which is accredited by the American Association for Accreditation of Laboratory Animal Care (AAALAC no. 000347) and has the Animal Welfare Assurance on file with the National Institutes of Health, Office of Laboratory Animal Welfare, OLAW assurance number A3205. All procedures employed were consistent with the Guide for the Care and Use of Laboratory Animals. The Institutional Animal Care and Use Committee of the George Washington University approved the protocol used for maintenance of mice and recovery of schistosomes.

### Method details

#### Computational search for gene safe harbors in *Schistosoma mansoni*

We undertook a genome analysis focusing of intergenic (gene-free) regions to identify prospective GSHs, using similar approaches as those used on the human genome.^28^ We aimed to locate a GSH, a site that would facilitate stable expression of the integrated transgene free of interference from the host genome and which, in parallel, integrates and transcribes the transgene without negative consequences or loss of fitness for the host cell. The search for GSHs deployed included several criteria, First, its location should be adjacent to peaks of H3K4me3, a histone modification associated with euchromatin and transcription start sites.^53^ Second, it should not be near or not containing H3K27me3 in any developmental stage, a histone mark associated with heterochromatin.^53^ Third, as the schistosome genome contains highly repetitive elements,^52^ the GSH site should be located in a unique tract of the genome sequence. Fourth, it should reside in open, euchromatic chromatin accessible to Tn5 transposase as assessed from ATAC-sequencing, which provides a positive display of transposase integration events^54^; consequently, safe harbor candidate regions should deliver an ATAC-sequence signal. Fifth, in the vicinity of known HIV integration sites, given that HIV integrates preferentially into euchromatin in human cells,^55^ we anticipated that HIV integration into the schistosome genome may likewise indicate a region of euchromatin (Figure 1A).^56^

To predict loci conforming to the criteria, pooled ChIP-seq data for H3K4me3 and K3K27me2 from previous studies were aligned against *S. mansoni* genome data (version 9 on the date of analysis). ATAC-seq was performed as described.^57^ Peakcalls of ChIP-seq and ATAC-Seq were done with ChromstaR^21^^,^^28^^,^^53^^,^^54^ and stored as Bed files. Bed files were used to identify the presence of H3K4me3 and absence of H3K27me3 in adults, miracidia, *in vitro* sporocysts, cercariae and *in vitro* schistosomula with bedtools intersect. Thereafter, ATAC-seq data from adult male and adult female worms (two replicates each) were intersected to find common ATAC-positive regions. H3K4me3-only (H3K27me3-absent) common to all stages and ATAC signals were intersected to find common regions. Next, the HIV integration sites were identified by using data from ERR33833.8. Reads were mapped to the lentivirus genome (HIV-1 vector pNL-3, accession AF324493.2) using Bowtie2 with default parameters. Paired reads were extracted where one end mapped to HIV and the other end mapped to schistosome genome at a unique location. Genes from the BED files above that located ≤11 kb HIV-1 integration sites were identified with bedtools closestbed. Gene expression data for these genes were obtained using the metanalysis tool, https://meta.schisto.xyz/analysis/, of Lu and Berriman.^44^

Computational searches that addressed these criteria predicted, *a priori*, gene free (intergenic)-GSH (Figure 1), given that transgene integration into an existing gene could diminish fitness of the genetically modified cell.^23^^,^^24^ We defined genes as protein coding sequences and sequences coding for long non-coding RNA (lncRNA). In view of our goal to use CRISPR/Cas mediated-HDR to insert the transgene, we searched preferentially for unique sequences, to obviate off-target gene modification, and excluded gene free-regions composed of repetitive sequences. Those unique sequences were also annotated outside lncRNA, regions beyond putative promotors that we deemed as 2 kb upstream of the transcription termination site (TTS), and the regions close to peaks of H3K4me2 in all parasite stages which never contained H3K27me3. The regions overlapping with ATAC-seq positive sites with ≤11 kb distance from HIV integration sites also were included. (The HIV-1 genome is ∼10 kb in length.) BEDtools were used to delimit 2 kb upstream regions (FlankBed). Annotations of 16,583 lncRNA were pooled from http://verjolab.usp.br/public/schMan/schMan3/macielEtAl2019/files/macielEtAt2019.bed12.^58^ Repeats were masked with RepeatMasker V4.1.0 using a specific repeat library produced with RepeatModeler2 V2.0.1 and stored as a GFF file. BED files with coordinates outside these annotations were generated by BedTools complementBed. BedTools Multiple Intersect was used to identify regions that are common to unique regions (complement of repeatmasker), intergenic regions, ≥2 kb upstream and outside of lncRNA. Regions which a length ≥100 bp were retained. These regions were intersected with merged H3K4me3-only common to all developmental stages and ATAC signals (euchromatic signal). BedTools ClosestBed was used to determine distance to the nearest integrated HIV provirus.

#### Schistosome egg culture

*S. mansoni* infected mice were euthanized six to seven weeks after infection, after which schistosomes were recovered by portal vein perfusion with 150 mM NaCl, 15mM sodium citrate, pH 7.0. The liver was resected, homogenized with a tissue blender, and the homogenate incubated with collagenase at 37°C for 18 h. Thereafter, schistosome eggs from the digested livers were recovered by Percoll gradient centrifugation, as described.^59^ Eggs isolated from livers, termed “liver eggs”, LE,^60^ were cultured in DMEM supplemented with 20% inactivated bovine serum, 2% antibiotic/antimycotic at 5% CO_2_, 37°C overnight before being subjected to transfection with the CRISPR materials.

#### Guide RNAs, ribonucleoprotein complexes

Here, we focused on GSH1, located on *S. mansoni* chromosome 3; 13231955–13233370 (Figure 1D), an intergenic safe harbor site of 1,416 nt, the longest in length of the four GSH (Figures 1B–1D). Guide RNAs (gRNA) for GSH1 were designed with the assistance of the CHOPCHOP^30^^,^^31^^,^^61^ tools, using the annotated *S. mansoni* genome,^62^ to predict target sites, off-targets, and efficiency of CRISPR/Cas9 programmed cleavage. CHOPCHOP predicted three overlapping guide RNAs targeting three cleavage sites within GSH1 with the three DSBs at six to 12 nt apart from each other. All three overlapping gRNAs lacked off-target sites and lacked self-complementarity. The three gRNAs were located on the forward strand of GSH1 at nucleotide positions 605–624, 617–636, and 623–642, respectively (Figure 2A). We termed the predicted DSBs as target 1, target 2 and target 3. Synthetic guide RNAs (sgRNA), specifically Alt-R CRISPR-Cas9 sgRNA chemically modified to enhance functional stability, and recombinant *Streptococcus pyogenes* Cas9 nuclease, specifically Alt-R HiFi Cas9, which includes nuclear localization sequences (NLS), were sourced from Integrated DNA Technologies, Inc. (IDT, Coralville, IA). Each ribonucleoprotein complex (RNP) was complexed in the separate tube, with Cas9 and a single sgRNA at 1:1 ratio in 25 μL Opti-MEM; 10 μL of 1 μg/μL sgRNA (Opti-MEM as diluent) was mixed with 10 μL of 1 μg/μL Cas9 (in Opti-MEM) by gentle pipetting and incubated for 10 min at 23°C to allow the RNP to assemble.

#### Doubled stranded DNA donor

A plasmid vector, pUC-Ubi-EGFP-ubi, was constructed by chemical synthesis of the donor transgene and its ligation into pUC (Azenta Life Sciences, Chelmsford, MA). The inserted sequence included homology arms of 600 bp length corresponding to GSH1 at 22–621 nt (5′-homology arm) and 640–1239 nt (3′-homology arm), respectively, flanking the in-frame expression cassette of the *S. mansoni* ubiquitin promoter (2,056 bp), EGFP (717 bp), and the ubiquitin terminator (578 bp). Plasmid DNA was amplified using Phusion High-Fidelity DNA Polymerase (New England Bio-Labs, Ipswich, MA, cat no. M0530) with primers specific for the 5′ and 3′ termini of the homology arms of 200, 400 or 600 bp in length (as primer list in Table S1). Thus, three forms of the donor transgene DNA were prepared, with homology arms of increasing length – 200, 400 and 600 bp. The primers employed to amplify the transgene DNA from the pUC-Ubi-EGFP-ubi plasmid were 5×phosphorothioate-modified to enhance stability of the amplified DNA. (5′-modified long dsDNA donor (lsDNA) enhances HDR and favors efficient single-copy integration by its retention of a monomeric conformation.^20^) (Figure 3A). PCRs were carried out in reaction volumes of 50 μL in 200 μM dNTPs, 0.5μM of each primer, 100 ng pUC-Ubi-EGFP-ubi, 3% DMSO and 1 unit of Phusion DNA polymerase, with thermocycling of 98°C, 30 s, 30 cycles of 98°C, 10 s, 55°C, 30 s, 72°C, 3 min, and final extension at 72°C, 10 min. Amplificons were isolated using the NucleoSpin Gel and PCR Cleanup and gel extraction kit (Takara, San Jose, CA, cat no. 740609), eluted in 30 μL nuclease-free water, and the long stranded (Ls) DNA donor transgene stored at −20°C until used.

#### Transfection of schistosome eggs

Ten thousand eggs (LE) of *S. mansoni* were washed three times with chilled (4°C) 1×PBS before transfer into a chilled electroporation cuvette (4 mm electrode gap, BTX, Holliston, MA) with Opti-MEM as the electroporation medium. Each 25 μL of RNP along with the lsDNA donor were immediately dispensed into the cuvette containing the schistosome eggs, to a total cuvette volume of 300 μL with Opti-MEM: specifically, for the dual guide RNA/RNPs, group 1) 25 μL RNP1+25 μL RNP2, group 2) 25 μL RNP2+25 μL RNP3, and group 3) 25 μL RNP1+25 μL RNP3. In groups with the lsDNA, 10 μg of this donor DNA was dispensed into the cuvette before bringing the final volume to 300 μL/cuvette. Transfection of schistosome eggs with CRISPR materials was undertaken using square wave electroporation (Electro SquarePorator ECM 830, BTX). Using a single pulse of 125 V for 20 ms^16^^,^^17^^,^^63^ was confirmed as optimal for use in this study based on analysis using higher voltages; viability as indicated by % miracidial hatching decreased progressively as voltage increased to 150, 200 and 250 V (Figure S3). The transfected eggs were transferred to culture medium, as above.

#### Nucleic acids

To recover genomic DNA and total RNA, eggs from each replicate were triturated in ∼100 μL DNA/RNA Shield solution (Zymo Research, cat no. R1100, Irvine, CA) using a motor-driven homogenizer fitted with a disposable pestle and collection tube (BioMasher II, Funakoshi, DiagnoCine LLC, NJ). DNA was isolated from 50% of the homogenate, and RNA from the remainder. 250 μL DNAzol ES (Molecular Research Center, Inc., Cincinnati, OH) was dispensed into the homogenate, and DNA recovered according to the manufacturer’s protocol. Total RNA was extracted from the homogenate by adding 250 μL RNAzol RT (Molecular Research Center, Inc.). Yields and purity were assessed quantified by spectrophotometry (NanoDrop One Spectrophotometer, Thermo Fisher Scientific), based on the ratios of absorbance at 260/280 and at 260/230 nm.^64^

#### Analysis of CRISPR on-target efficiency

Amplicons of GSH1 spanning the programmed DSBs were obtained using population genomic DNA (above) and primers termed ‘indel-F and indel-R primers’ that cover the region flanking expected double-strand break of all the CRISPR target sites (Figure 2A, Table S1). Amplification products were purified (NucleoSpin Gel and PCR Cleanup and gel extraction kit, Takara; cat no. 740609) and the nucleotide sequences determined by Sanger cycle sequencing (Azenta Life Sciences, South Plainfield, NJ). Chromatograms of the sequence traces of experimental and control group(s) was compared using DECODR^32^ at default parameters with single and multiple CRISPR target analysis (Figures 2B–2F and S1A). The pattern of indels in was confirmed by TIDE analysis of Sanger sequence reads^65^ (Figures S1B–S1E).

#### Detection of integration of the transgene into the schistosome genome

Integration of the donor transgene at GSH1 was analyzed by PCR, using GoTaq G2 DNA polymerase (Promega, Madison, WI) and two pairs of primers; one primer located on the GSH1 using specific primers upstream (5′ KI-F) or downstream (3′ KI-R) of the homology arms paired with primers specific for the transgene (5′ KI-R or 3′ KI-F) (Figure 2B, Table S1), as described.^17^ The PCR cycling conditions were 95°C, 2 min, 40 cycles 94°C, 15 s, 58°C 30 s, 72°C, 60 s, and the amplification products were separated by electrophoresis and stained with ethidium bromide. The expected product sizes for the 5 ′ and 3′ integration site-specific amplicons were 728 and 983 bp, respectively, and the amplification control (amplified by indel-F and indel-R primers), expected product size, 764 bp (Figure 2C).

### Quantification and statistical analysis

#### Quantification of transgene expression in schistosome eggs

To examine the mRNA expression of EGFP, total RNAs extracted from LE were exposed to DNase to eliminate residual genomic DNAs and donor lsDNA donor were transcribed into cDNA using the Maxima First Strand cDNA synthesis kit with DNase (Thermo Fisher Scientific). The qPCR was performed using the GoTaq G2 DNA polymerase (cat no. M7841, Promega, Madison, WI) with the specific primers; EGFP-F and EGFP-R (Figure 3B, Table S1) with expected amplicon at 717 bp. *S. mansoni* GAPDH (Smp_056970) served as the reference gene. The specific primer for GAPDH Table S1) expected amplicon of 285 bp in length. PCR cycling conditions: 95°C, 2 min, 25 cycles 94°C, 15 s, 58°C, 30 s, 72°C, 30 s, after which amplification products were examined, as above.

#### Quantification of fluorescence by spectral imaging and linear unmixing

Spectral and spatial distribution of EGFP fluorescence were assessed using confocal laser scanning microscopy, using a Zeiss LSM710 Meta detector fitted Axiovert 200 (Carl Zeiss, Jena, Germany). Images were collected with the W Plan-Apochromat 20×/1.0 NA water immersion objective. Spectroscopic measurements were performed in response to excitation by 458 nm (16.5 μW) Argon laser line and 633 nm He/Ne laser line (Lasos Lasertechnik, Jena, Germany), which were used for focus and transmission mode imaging. Emission was detected with a spectral META detector at 16 channels, scanning at 477 nm through 638 nm, simultaneously. A hurdle for the detection of EGFP using fluorescence microscopy is presented by the autofluorescence known to originate from the schistosome eggshell,^66^^,^^67^^,^^68^ with vitelline cells the likely origin of this emission.^69^ To surmount this hurdle, the EGFP spectrum emitted from within each eggshell was investigated by selecting the area to be examined, specifically the entire miracidium, and collecting multispectral images of the miracidium within the eggshell using the LSM Image Examiner. The images collected were assessed for EGFP by subtracting regions emitting autofluorescence from the fluorescence signal collected for entire surface area of the egg: Specifically, the total EGFP intensity at 509 nm^69^ was calculated using the Zeiss Zen (black edition) software module from ∼400 eggs (∼100 eggs from each of four biological replicates) in each of the control and experimental groups.

Zen Blue software (Zeiss) was used to measure EGFP fluorescence intensity in confocal micrographs of schistosome eggs. Prism version 9 was used in analysis of these data and to establish mean and standard deviation and levels of statistical significance which are shown in figures. Exact values of n are provided in relevant figure legends.

## Data Availability

• The nucleotide sequence reads are available at the NIH Sequence Read Archive, BioProject PRJNA919068, accession numbers SRX18957908-18957932. BED files from the bioinformatics analysis are publicly available on Zenodo. The DOI is https://zenodo.org/record/7602535#.ZAIgqBPMLIM. It is also listed in the key resources table. • This paper dose not report original code. • Any additional information required to reanalyze the data reported in this paper is available from the lead contact upon request.

## Supplemental information

Document S1. Figures S1–S3 and Table S1

Document S2. Article plus supplemental information
