# Supplementary material for: Targeted insertion and reporter transgene activity at a gene safe harbor of the human blood fluke, Schistosoma mansoni
Source: Cell Rep Methods. 2023 Jul 24;3(7):100535. doi: 10.1016/j.crmeth.2023.100535 (PMC10391569; doi:10.1016/j.crmeth.2023.100535)
Supplement: Document S1. Figures S1–S3 and Table S1 [file mmc1.pdf]

Cell Reports Methods, Volume *3*

Supplemental information

Targeted insertion and reporter transgene activity at a gene safe harbor of the human blood fluke, *Schistosoma mansoni*

Wannaporn Ittiprasert, Max F. Moescheid, Cristian Chaparro, Victoria H. Mann, Thomas Quack, Rutchanee Rodpai, André Miller, Prapakorn Wisitpongpun, Watunyoo Buakaew, Margaret Mentink-Kane, Sarah Schmid, Anastas Popratiloff, Christoph G. Grevelding, Christoph Grunau, and Paul J. Brindley

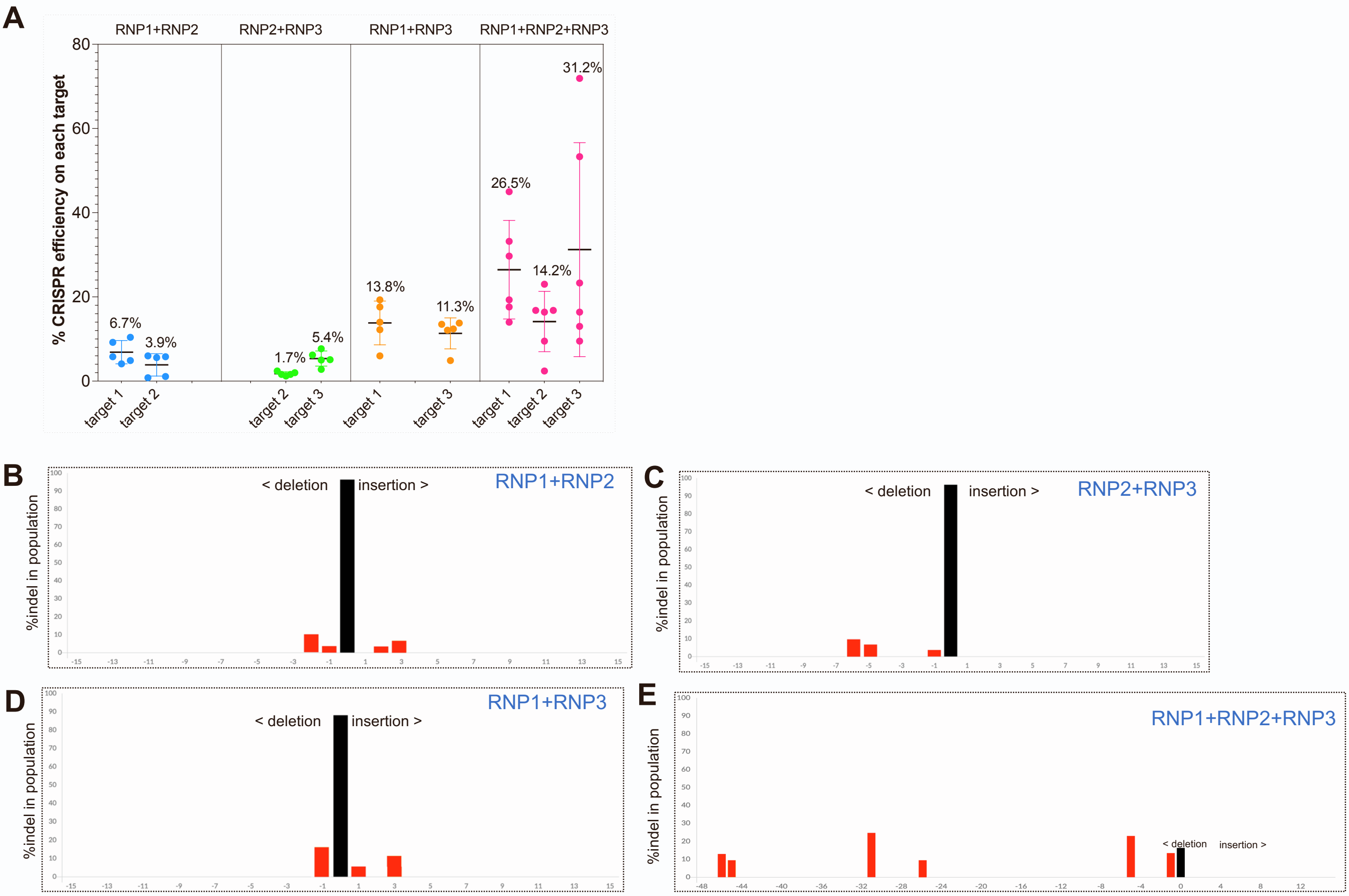

Figure S1. Percentage of CRISPR efficiency, related to Figure 2.

A) CRISPR efficiency (percentage) on individual target sites as estimated using the Deconvolution of Complex DNA Repair (DECODR) algorithm using distance from dual gRNAs; RNP1+RNP2 (blue dots), RNP2+RNP3 (green), RNP1+RNP3 (yellow) and triple gRNAs; RNP1+RNP2+RNP3 (pink dots), plotted from 5-6 independent biological replicates. The % indels on each target varied among RNP combinations: transfection with triple RNPs lead to highest CRISPR efficiencies on each target site 1, 2 and 3, 26.5%, 14.2% and 31.2%, respectively, compared with the dual RNP groups. The % indels resulting from dual RNPs on target 1 were 6.7% and 13.8%; target 2, 3.9% and 1.7%, and target 3, 5.4% and 11.3%. **B) to E)**, examples of indel patterns resulting from the RNPs. DECODR software plots confirmed the indel profiles and were similar to those obtained with TIDE (Figure S1B to S1E).

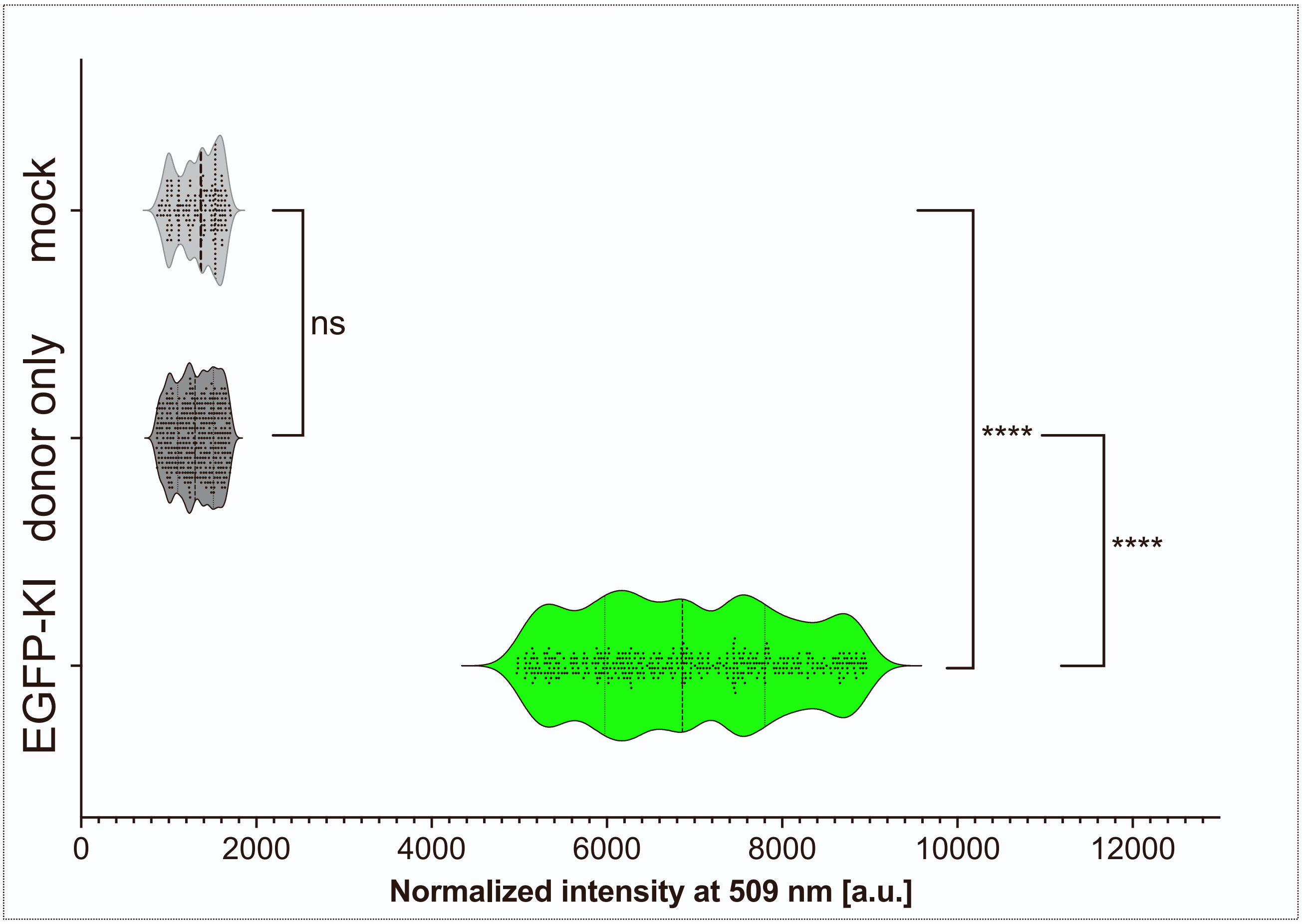

Figure S2. **High EGFP fluorescent intensity from KI egg, related to Figure 4.**

Emission spectral intensity for eggs, scanned from 477 to 638 nm, normalized fluorescence spectral intensity from control eggs (transfected with donor repair template) exhibiting higher intensity than autofluorescence; these eggs were also scored as EGFP-positive, and with a normalized EGFP intensity mean, 1290 au (range, 856-1713); experimental group, normalized-EGFP intensity, mean 6905 au (range 4972 – 8963); $P < 0.001$, unpaired *t*-test, $n = 402$; difference between means of experimental and control group eggs ± SEM, 5651 ± 57.4, 95% CI, 5502 to 5728).

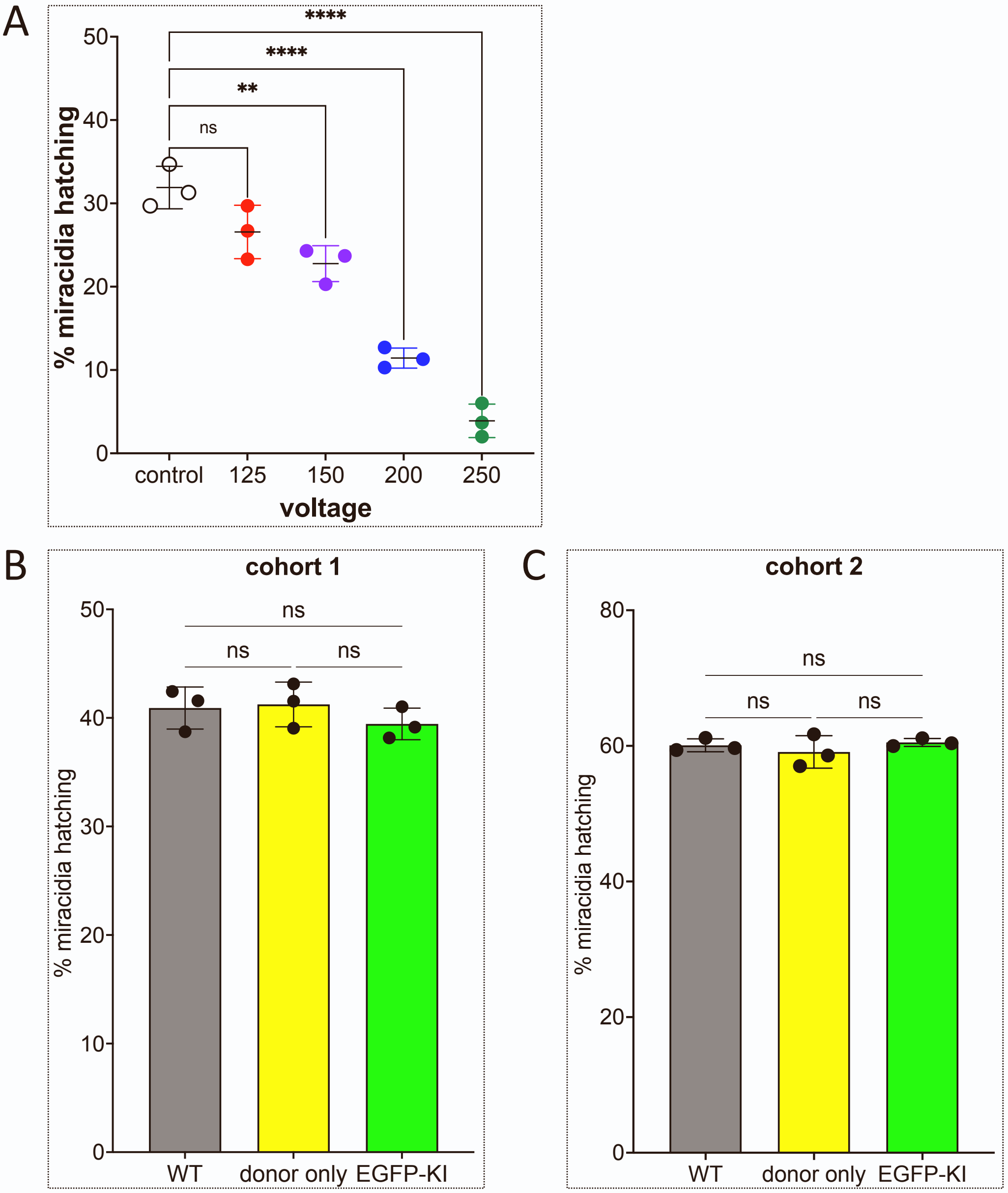

Figure S3. Investigation of miracidial hatching , related to STAR METHODS.

A) percent miracidia hatched following transfection by electroporation of multiple RNPS at each of 125, 150, 200 and 250 volts. Survival and/or larval growth inside the egg in the 125 volts group was not significantly affected in comparison to the control group; 26.6±3.2%, 125 V group, 31.9±2.6%, control, non-electroporated group. By contrast, hatching progressively decreased as voltage increased: 150 V, 22.8±2.2%; 200 V, 11.4±1.2%; 250 V, 3.9±2.0%, respectively ($P \leq 0.001$, two-way ANOVA). **B)** and **C)** differences were not seen in hatching rate among the treatments group of the control (WT), donor only and EGFP KI treatment groups. The experiment was performed twice with a different cohort of schistosome eggs each time, here termed cohort 1 (**B**) and cohort 2 (**C**). In both panels B and C, each treatment included two technical replicates, each replicate of ~1,000 eggs. Hatching rate was established based on three blinded counts for each replicate.

Table S1. Primer list and nucleotide sequences used in PCRs to amplify DNA donor, investigate EGFP transcript, programmed knockout and knock-in, related to STAR Methods

Primer	5' to 3' sequence
Indel-F	TGTTATCGTCCGTCGCTTCA
indel-R	GCGTTCAAACATTGCCCACT
600 bp HA-F	AGATTGCTAGAAATTTATGAAAG
600 bp HA-R	ACTGCCGAATTTATAATATTTGG
400 bp HA-F	GATGAGTGCATCGATCGATTAC
400 bp HA-R	GAATGATGTTGGAAGTCAGA
200 bp HA-F	CGCTTCAATATTGTTTTTTGC
200 bp HA-R	CACACCTTCAAAACAATGTTTC
EGFP-F	ATGGTGAGCAAGGGCGAGG
EGFP-R	CTTGTACAGCTCGTCCATGCC
SmGAPDH-F	ATGGGACATTTCCAGGCGAG
SmGAPDH-R	CCAACAACGAACATGGGTGC
5' KI-F	AGGGTTTTGGTTCGCAGGAT
5' KI-R	CGGAGACAATCTGGAAAGGTCA
3' KI-F	GTCCGCGTAATCGTCGTTACTA
3' KI-R	GTGGTTCCATACTATGCAGTTTCC
